# Comparison of nutrients and ultra-processed food consumption between different phenotypes defined by abdominal obesity and sarcopenia

**DOI:** 10.3389/fnut.2025.1683196

**Published:** 2026-01-19

**Authors:** Eunjin Jang, Sarang Jeong, Jinhyun Kim, Sukyoung Jung, Jee Young Kim, Jung Eun Lee, Sohyun Park, Jang Won Son, On behalf of the GOMS study group, Korean Society for the Study of Obesity

**Affiliations:** 1 The Korean Institute of Nutrition, Hallym University, Chuncheon, Republic of Korea; 2 Department of Food Science and Nutrition, Hallym University, Chuncheon, Republic of Korea; 3 Department of Healthcare Policy Research, Korea Institute for Health and Social Affairs, Sejong, Republic of Korea; 4 Jeju Jinsan Company, Seogwipo, Republic of Korea; 5 Department of Food and Nutrition, College of Human Ecology, Seoul National University, Seoul, Republic of Korea; 6 Research Institute of Human Ecology, College of Human Ecology, Seoul National University, Seoul, Republic of Korea; 7 Division of Endocrinology and Metabolism, Department of Internal Medicine, Bucheon St. Mary’s Hospital, College of Medicine, The Catholic University of Korea, Seoul, Republic of Korea

**Keywords:** obesity, sarcopenia, sarcopenic obesity, nutrients, ultra-processed food

## Abstract

**Background:**

Obesity, sarcopenia, and sarcopenic obesity require effective nutritional strategies in public health.

**Methods:**

This cross-sectional study analyzed 535 adults aged 40–60 years in rural Korea. Participants were grouped by abdominal obesity (waist circumference ≥90 cm for men, ≥85 cm for women) and probable sarcopenia (handgrip strength ≤28 kg for men, ≤18 kg for women). Dietary intake was assessed using a semi-quantitative food frequency questionnaire and NOVA classification. Associations between food group intake and each phenotype were analyzed using multinomial logistic regression, adjusting for confounders.

**Results:**

Compared with healthy controls, the probable sarcopenia group reported lower intakes of total protein (*p* = 0.041), vitamin A (*p* = 0.041), and carotenoids (*p* = 0.046), and higher intake of processed culinary ingredients (*p* = 0.012). The sarcopenic obesity group had lower intake of minimally processed foods (*p* = 0.046) and higher intake of ultra-processed foods (*p* = 0.010). In regression models, higher protein intake was associated with lower odds of probable sarcopenia (OR = 0.73, 95% CI: 0.56–0.95). Higher ultra-processed food intake was associated with increased odds of abdominal obesity (OR = 1.26, 95% CI: 1.03–1.54) and sarcopenic obesity (OR = 1.37, 95% CI: 1.02–1.84).

**Conclusion:**

Higher protein and minimally processed food intakes were associated with lower odds of probable sarcopenia, whereas higher ultra-processed food intake was associated with higher odds of abdominal and sarcopenic obesity in middle-aged adults in rural Korea. These observational findings may inform hypothesis generation and public health planning, but prospective studies are needed to confirm these associations and assess causality.

## 1 Introduction

The prevalence of abdominal obesity has shown a steady increase across all age groups in Korea, reaching 38.4% in 2021 (1). This trend is particularly concerning as abdominal obesity is strongly associated with an elevated risk of various chronic diseases. Korean studies have reported significant links between abdominal obesity and the incidence of hypertension, dyslipidemia, and diabetes in middle-aged and older adults (2). Additionally, individuals with abdominal obesity have a higher relative risk of developing type 2 diabetes, myocardial infarction, and certain cancers compared to those with normal weight (3), and older adults with abdominal obesity are more likely to experience frailty (4).

Changes in body composition, characterized by increased fat mass and decreased muscle mass, are an inherent aspect of the aging process and significantly impact the health of older adults (5, 6). Sarcopenia, an age-related condition characterized by progressive loss of muscle mass, decreased muscle strength, and impaired muscle function, poses a significant public health concern among middle-aged and elderly populations (7). Research has consistently demonstrated the association of sarcopenia with various health conditions, including an increased prevalence of metabolic syndrome (8), functional impairment and physical disability (9), increased risk of falls and fractures (10), as well as a higher risk of frailty and mortality (10, 11).

Sarcopenic obesity, characterized by low muscle mass, muscle quality, or strength combined with high fat mass, has emerged as a major health concern among older adults, representing a new form of obesity in the elderly population (12). Due to the increased metabolic burden caused by both sarcopenia and obesity, sarcopenic obesity plays a significant role in cardiometabolic risk (13). Research has demonstrated that individuals with sarcopenic obesity are at a higher risk of cardiovascular diseases (14) and mortality compared to individuals without sarcopenia (15). Furthermore, individuals with sarcopenic obesity were found to have a higher risk of cardiovascular diseases, diabetes, and physical dysfunction compared to those with either sarcopenia or obesity alone (16). Both sarcopenia and sarcopenic obesity are key factors that hinder healthy aging and are closely linked to quality of life (17, 18).

Obesity, sarcopenia, and sarcopenic obesity have emerged as major health concerns in modern society, with dietary factors playing a crucial role in their development and progression. Existing research suggests that obesity results from an imbalance between energy intake and expenditure (19), while inadequate protein intake and nutritional imbalances are recognized as primary contributors to sarcopenia and sarcopenic obesity. A high-protein diet is particularly essential for maintaining muscle mass and function, with substantial research focused on the impact of protein intake in sarcopenia prevention. Additionally, specific nutrients, such as vitamin D and omega-3 fatty acids, have received considerable attention for their effects on muscle health and body fat regulation (20).

Recently, dietary patterns based on the level of food processing have gained attention. The NOVA classification categorizes foods into minimally processed foods (MPF, NOVA1), processed culinary ingredients (PCI, NOVA2), processed foods (PF, NOVA3), and ultra-processed foods (UPF, NOVA4) (21). While MPF intake is generally associated with better dietary quality (21) high consumption of PCI, such as salt and sugar, can indirectly reduce dietary quality by displacing nutrient-dense foods (22, 23). PF may exert neutral or beneficial metabolic effects depending on their composition, as observed with certain items like cheese (24). In contrast, high UPF intake has been linked to increased risks of frailty (25), decline in grip strength (26), and muscle mass loss (27). However, no studies have comprehensively investigated associations between intakes of all four NOVA groups and obesity and sarcopenia among middle-aged and older Korean adults. Therefore, dietary factors related to obesity, sarcopenia, and sarcopenic obesity should be examined more comprehensively by considering the level of food processing.

Sarcopenia is associated with increased risks of falls, fractures, functional decline, and mortality, and is often left undiagnosed, highlighting the importance of early detection and prevention (28, 29). Probable sarcopenia (PS), defined by reduced handgrip strength without assessments of muscle mass or physical performance, has recently gained attention as a practical early marker of sarcopenia risk. According to the European Working Group on Sarcopenia in Older People (EWGSOP), low muscle strength is the most critical determinant of sarcopenia, and early intervention is essential once strength decline is observed (30). Although most sarcopenia studies have focused on older adults aged 60 and over, muscle strength tends to decline from midlife. Therefore, studies that include middle-aged adults are essential for informing early prevention strategies. PS is particularly suitable for assessing sarcopenia risk in both middle-aged and older populations and may serve as an effective tool for early identification and management.

Gangwon Province in South Korea is a super-aged region where more than 20 percent of the population is aged 65 years or older, and its terrain is largely mountainous and rural (31). According to the 2022 Community Health Survey, the province has the second-highest obesity rate in the country (32). In response, the Gangwon Obesity and Metabolic Syndrome (GOMS) study was launched to investigate the factors contributing to the high obesity prevalence and to support region-specific public health strategies (33). Using data from the GOMS cohort, the present study aimed to assess the prevalence of abdominal obesity and PS in adults aged 40 to 60 years and to examine associated dietary factors, with the goal of providing evidence to support early prevention efforts targeting rural populations.

## 2 Methods

### 2.1 Study design and participants

This was a cross-sectional study utilizing baseline survey data from the GOMS cohort. The GOMS cohort is an ongoing community-based prospective cohort study designed to assess body weight and body composition status among middle-aged and older adults residing in rural areas of the Gangwon Province, investigating obesity-related factors, and examining longitudinal changes in obesity (33). The study participants consisted of Korean residents aged 20–69 years living in the Yeongseo region of Gangwon Province. The exclusion criteria comprised illiterate individuals, those with moderate to severe cognitive impairment, and those currently participating in other observational or clinical studies. The first-year baseline survey was conducted from June to July 2022, recruiting 319 articipants, while the second survey was conducted from September to October 2023, recruiting 317 participants. After excluding individuals with missing key measurements, such as grip strength, a total of 535 participants (130 males and 405 females) aged 40–60 was included in the final analysis. This study was approved by the Institutional Review Board of the authors’ institution.

### 2.2 Data collection procedures

The data collection process was conducted by trained researchers and research assistants. Before data collection, experienced researchers conducted two training sessions, comprising both theoretical instruction and hands-on practice. The training covered all aspects of the survey, including direct practice in anthropometric measurements and grip strength assessments. To minimize measurement errors, simulations were employed for challenging measurements. Additionally, study participants received presurvey materials to facilitate their understanding of the survey procedures.

Upon arrival at the designated survey site, participants received a detailed explanation of the study’s purpose and procedures, followed by the obtention of written informed consent. Data collection included anthropometric measurements and grip strength assessments. Additionally, participants completed a self-administered questionnaire, which gathered information on demographic characteristics, physical activity levels, alcohol consumption habits, smoking status, and dietary intake patterns. To ensure data accuracy, trained researchers conducted follow-up interviews to review the completed questionnaires, addressing any missing responses or correcting inaccurate answers.

### 2.3 Dietary assessment

#### 2.3.1 Includes macronutrient and micronutrient intake, and NOVA-based food classification

Dietary intake was assessed using a semiquantitative Food Frequency Questionnaire (FFQ), which was developed and validated through the Korea National Health and Nutrition Examination Survey (KNHANES) to evaluate the habitual dietary intake of Korean adults (34, 35). The FFQ consists of 112 food items, and participants reported their consumption frequency and portion size for each item over the past year. The frequency categories were divided into nine levels (never or rarely, once per month, two to three times per month, once per week, two to four times per week, five to six times per week, once per day, twice per day, and three times per day). Portion sizes were categorized as small, medium, and large (34). Participants with daily caloric intakes outside the plausible range (less than 500 kcal or more than 5,000 kcal) were excluded from the study to ensure data validity.

Dietary data obtained from the FFQ were categorized using the NOVA food classification system, which groups foods according to the extent and purpose of industrial processing (36). Because the NOVA system was originally developed based on Brazilian dietary patterns, its direct application to Korean mixed dishes is limited. Therefore, this study referred to the Korean-adapted NOVA classification framework developed to reflect the Korean food environment (37) and applied the FFQ-based NOVA classification method validated for use with FFQ data (38). This method demonstrated good agreement between FFQ-based and 24-h recall-based estimates of UPF intake using data from the KNHANES 2016, confirming the applicability of the NOVA classification to FFQ data in Korean adults. Based on this validated framework, 109 FFQ items (excluding three alcohol-related items) were classified into the four NOVA groups according to the item-specific categorization matrix presented by Jung et al. (38). The contribution of each NOVA group to total energy intake was calculated as a percentage of total daily caloric intake. Detailed classification of all FFQ items into the four NOVA groups is provided in Supplementary Table S2.

### 2.4 Anthropometric and muscle strength measurement

#### 2.4.1 Includes abdominal obesity and muscle strength assessments

Body mass index (BMI) has limitations in assessing obesity in older adults, as it does not account for age-related muscle loss (39). In contrast, abdominal obesity, measured by waist circumference (WC), is a more sensitive indicator of visceral fat accumulation and is a better predictor of the risk of metabolic syndrome and cardiovascular diseases in middle-aged and older adults (40, 41). In this study, trained examiners measured WC to the nearest 0.1 cm using a measuring tape at the midpoint between the lower rib margin and the iliac crest. Abdominal obesity was defined according to the Korean Society for the Study of Obesity (KSSO) criteria, with cutoff values of ≥90 cm for males and ≥85 cm for females (42).

The diagnosis of sarcopenia typically involves a comprehensive assessment of muscle strength, muscle mass, and physical function. This study focused on middle-aged and older adults aged 40–60, emphasizing the importance of early detection and preventive intervention for sarcopenia. To identify individuals at risk, the Asian Working Group for Sarcopenia (AWGS) criteria for PS were applied (43). PS is a method for screening sarcopenia risk solely based on muscle strength, without evaluating muscle mass or physical function. According to AWGS guidelines, PS is defined as a grip strength of ≤28 kg for males and ≤18 kg for females (43). Grip strength was measured using a digital hand dynamometer (TKK-5401; TAKEI, Tokyo, Japan) following standardized procedures. Participants were instructed to stand upright with their wrist and arm in a neutral position, and their elbow fully extended. Grip strength was measured once on each hand, and the average value was used as the final muscle strength score. To ensure consistency in measurement conditions, all participants received detailed instructions on proper posture and exerting maximum effort before the assessment.

### 2.5 Phenotype classification and statistical analysis

#### 2.5.1 Defines phenotypes and details statistical methods used

Participants were classified into four groups based on the AWGS-defined PS criteria and the KSSO-defined abdominal obesity criteria. The healthy control group consisted of individuals who did not meet the criteria for either PS or abdominal obesity. The obesity group comprised participants who had abdominal obesity but did not meet the PS criteria. The PS group comprised individuals who met the PS criteria but did not have abdominal obesity. Lastly, the sarcopenic obesity group consisted of participants who met both the PS and abdominal obesity criteria. For the purpose of this study, the term sarcopenia is used interchangeably with PS, as sarcopenic obesity is defined as the coexistence of PS and abdominal obesity. By classifying participants based on abdominal obesity and PS status, this study aimed to identify the risk stages of sarcopenia, focusing on muscle weakness, and conduct an in-depth analysis of its interaction with abdominal obesity.

#### 2.5.2 Covariates

The key covariates comprised biological (gender, age), socioeconomic (education level, household income), and lifestyle domains (drinking, smoking, physical activity). The main covariates included age, gender, education level, household income, drinking, smoking, and physical activity. Age was used as a continuous variable, and education level was classified into middle school graduation or lower, high school graduation, and university graduation or higher. Household income was classified into less than 2 million Korean won, 2 to 4 million won, and more than 4 million won based on monthly household gross income. Drinking was defined as the current drinking status (yes or no), and smoking was classified into non-smoking (less than 100 cigarettes in a lifetime), past smoking, and current smoking. Physical activity was classified into three categories: no physical activity, insufficient or inactive physical activity, and recommended physical activity. Recommended physical activity was defined according to standard guidelines as engaging in at least 150 min of moderate-intensity activity or 75 min of vigorous-intensity activity per week (44). In addition, minutes of vigorous-intensity activity were counted as double when combined with moderate-intensity activity. Based on this, whether physical activity was satisfied was classified into no physical activity, not meeting physical activity, and met physical activity.

#### 2.5.3 Statistical analysis

Descriptive statistical analysis was performed to present the general characteristics of the subjects and the ratio or average of the outcome variables. In addition, the relationship between abdominal obesity and sarcopenia and dietary factors was analyzed after correcting for major covariates using multiple regression analysis. To compare differences between groups, dummy variables were created for group classification, and multiple regression analysis was performed with the healthy control group as the reference. Post-estimation analyses, including hypothesis tests and linear combination tests, were conducted to assess statistical differences between the healthy control group and other groups. Additionally, multinomial logistic regression models were used to estimate covariate-adjusted odds ratios (ORs) and 95% confidence intervals (CIs) for abdominal obesity, PS, and sarcopenic obesity, using the healthy control group as the reference. Quartiles 2, 3, and 4 of each dietary factor were compared with quartile 1. All analyses were performed using Stata MP 17.0 (Stata Corp LLC, College Station, TX, USA).

## 3 Results

The general characteristics of participants by obesity and PS status are presented in Table 1. The mean age was 56.0 years, and the proportion of females was higher (75.7%, *p* = 0.013). The age distribution was 24.3% aged 40–49 years, 38.1% aged 50–59 years, and 37.6% aged 60–69 years (*p* = 0.004). The PS group showed a higher proportion of individuals in their 50s, whereas the obesity and sarcopenic obesity groups showed relatively higher proportions of individuals in their 60s. The obesity and sarcopenic obesity groups showed the lowest proportion with a college education or higher. The sarcopenic obesity group showed a relatively higher proportion with income <2 million KRW. No statistically significant between-group differences were observed for smoking status, alcohol consumption, or physical activity.

**Table 1:** General characteristics of the study participants by four phenotypes defined by abdominal obesity and probable sarcopenia.

Variable	Healthy control^a^	Central obesity^b^	Probable sarcopenia^c^	Sarcopenic obesity^d^	Total	*p*-value^*^
*N* (%)	239 (44.7)	176 (32.9)	70 (13.1)	50 (9.3)	535 (100.0)	
Sex						0.013
Male	63 (26.4)	51 (29.0)	8 (11.4)	8 (16.0)	130 (24.3)	
Female	176 (73.6)	125 (71.0)	62 (88.6)	42 (84.0)	405 (75.7)	
Age						0.004
40–49	77 (32.2)	31 (17.6)	16 (22.9)	6 (12.0)	130 (24.3)	
50–59	86 (36.0)	67 (38.1)	30 (42.9)	21 (42.0)	204 (38.1)	
60–69	76 (31.8)	78 (44.3)	24 (34.3)	23 (46.0)	201 (37.6)	
Marital status (*N* = 532)						0.751
Married^e^	193 (81.1)	144 (82.8)	56 (80.0)	38 (76.0)	431 (81.0)	
Not married^f^	45 (18.9)	30 (17.2)	14 (20.0)	12 (24.0)	101 (19.0)	
Education level (*N* = 532)						<0.001
≤Middle school	52 (21.8)	67 (38.1)	18 (25.7)	20 (40.0)	157 (29.4)	
High school	96 (40.2)	76 (43.2)	34 (48.6)	22 (44.0)	228 (42.6)	
≥College	91 (38.1)	33 (18.8)	18 (25.7)	8 (16.0)	150 (28.0)	
Household income per month (*N* = 532)						0.001
<2 million Won	50 (21.0)	52 (29.7)	14 (20.3)	22 (44.0)	138 (25.9)	
2–4 million Won	85 (35.7)	59 (33.7)	34 (49.3)	19 (38.0)	197 (37.0)	
>4 million Won	103 (43.3)	64 (36.6)	21 (30.4)	9 (18.0)	197 (37.0)	
Smoking status (*N* = 529)						0.275
Never smoker^g^	184 (78.0)	130 (75.1)	59 (84.3)	39 (78.0)	412 (77.9)	
Former smoker	21 (8.9)	27 (15.6)	6 (8.6)	6 (12.0)	60 (11.3)	
Current smoker	31 (13.1)	16 (9.3)	5 (7.1)	5 (10.0)	57 (10.8)	
Drinking status (*N* = 534)						0.290
Current drinker	161 (67.4)	122 (69.7)	43 (61.4)	24 (48.0)	350 (65.5)	
Current abstainer	78 (32.6)	53 (30.3)	27 (38.6)	26 (52.0)	184 (34.5)	
Recommended PA levels						0.650
No PA	131 (54.8)	97 (55.1)	43 (61.4)	33 (66.0)	304 (56.8)	
Insufficient or inactive PA	37 (15.5)	32 (18.2)	11 (15.7)	5 (10.0)	85 (15.9)	
Recommended PA^h^	71 (29.7)	47 (26.7)	16 (22.9)	12 (24.0)	146 (27.3)	

PA, physical activity. Probable sarcopenia is interchangeably referred to as sarcopenia in this study.

*p-values were calculated using the chi-square test for categorical variables and ANOVA for continuous variables.

a Healthy control: participants without central obesity and sarcopenia.

b Central obesity: waist circumference ≥90 cm for males and ≥85 cm for females.

c Probable sarcopenia: handgrip strength ≤28 kg for males and ≤18 kg for females.

d Sarcopenic obesity: meeting both criteria for central obesity and probable sarcopenia.

e Married: includes common-law partnerships.

f Not married: includes individuals who are separated, divorced, widowed, or single.

g Never smoker: fewer than 100 cigarettes in a lifetime.

h Recommended PA levels: ≥150 min/week of moderate-intensity or ≥75 min/week of vigorous-intensity PA.

Table 2 summarizes differences in daily macro- and micronutrient intake by obesity and PS status. Compared with the healthy control group, the PS group showed lower mean intakes of total protein (*p* = 0.041), vitamin A (*p* = 0.041), and carotenoids (*p* = 0.046). No other nutrients differed significantly.

**Table 2:** Daily nutrient intake by four phenotypes defined by abdominal obesity and probable sarcopenia.

Variable	Healthy control^a^	Central obesity^b^	Probable sarcopenia^c^	Sarcopenic obesity^d^	*p*-value
*N*	239	176	70	50	
Total energy (kcal/day)	1,980.0 (55.3)	1,964.9 (65.1)	2,058.4 (102.4)	1,910.3 (118.0)	0.803
Carbohydrates (%kcal/day)	59.4 (0.7)	59.6 (0.9)	61.8 (1.4)	58.4 (1.6)	0.345
Total protein (%kcal/day)	14.7 (0.2)	14.5 (0.3)	13.7 (0.4)*	14.9 (0.5)	0.179
Fat (%kcal/day)	20.9 (0.5)	21.0 (0.6)	20.1 (0.9)	21.9 (1.1)	0.661
Saturated fatty acids (% kcal/day)	6.1 (0.2)	6.1 (0.2)	5.9 (0.3)	6.5 (0.3)	0.609
Monounsaturated fatty acids (%kcal/day)	6.6 (0.2)	6.6 (0.2)	6.4 (0.3)	7.0 (0.4)	0.670
Polyunsaturated fatty acids (%kcal/day)	5.4 (0.1)	5.5 (0.2)	5.0 (0.3)	5.4 (0.3)	0.427
Omega-3 fatty acids (%kcal/day)	0.6 (0.0)	0.7 (0.0)	0.6 (0.0)	0.6 (0.0)	0.252
Omega-6 fatty acids (%kcal/day)	4.9 (0.1)	5.0 (0.2)	4.5 (0.2)	4.9 (0.3)	0.429
Cholesterol (mg/day)	332.3 (12.2)	318.9 (14.4)	330.5 (22.6)	322.3 (26.1)	0.908
Dietary fiber (g/day)	21.7 (0.5)	22.7 (0.6)	20.9 (1.0)	21.2 (1.2)	0.400
Potassium (mg/day)	2,925.3 (62.8)	2,929.9 (73.9)	2,844.1 (116.4)	2,732.2 (134.1)	0.556
Calcium (mg/day)	487.2 (12.8)	476.8 (15.1)	447.0 (23.8)	485.1 (27.4)	0.514
P (mg/day)	1,060.8 (15.1)	1,044.5 (17.7)	1,018.4 (27.9)	1,043.2 (32.2)	0.596
Iron (mg/day)	14.6 (0.3)	14.7 (0.3)	13.6 (0.5)	14.0 (0.5)	0.153
Sodium (mg/day)	3,375.1 (85.8)	3,523.7 (101.0)	3,153.8 (159.1)	3,570.1 (183.3)	0.193
Vitamin A (μg RE/day)	673.6 (23.2)	661.4 (27.3)	567.3 (43.0)*	602.5 (49.6)	0.127
Retinol (μg /day)	104.9 (3.8)	97.4 (4.4)	95.7 (7.0)	100.3 (8.0)	0.519
Carotenoid (μg/day)	3,270.2 (130.4)	3,269.8 (153.5)	2,720.6 (241.8)*	2,906.1 (278.5)	0.154
Thiamine (mg/day)	1.9 (0.0)	1.9 (0.0)	1.9 (0.0)	1.9 (0.1)	0.819
Riboflavin (riboflavin, mg/day)	1.5 (0.0)	1.4 (0.0)	1.4 (0.1)	1.4 (0.1)	0.800
Niacin (mg/day)	14.0 (0.2)	13.7 (0.3)	13.1 (0.4)	13.6 (0.5)	0.346
Vitamin C (mg/day)	113.1 (5.7)	110.7 (6.7)	106.4 (10.6)	89.2 (12.2)	0.363

PA, physical activity. Probable sarcopenia is interchangeably referred to as sarcopenia in this study.

*p < 0.05 compared with the healthy control group.

Data are presented as mean (standard deviation). Means were estimated using multiple regression analysis with the healthy control group as the reference, adjusting for age, sex, education level, household income, alcohol consumption, smoking status, and meeting recommended PA levels. *p*-values represent overall group differences from the same model. Total energy intake was adjusted for all covariates except those expressed as %kcal/day.

a Healthy control: participants without central obesity and sarcopenia.

b Central obesity: waist circumference ≥90 cm for males and ≥85 cm for females.

c Probable sarcopenia: handgrip strength ≤28 kg for males and ≤18 kg for females.

d Sarcopenic obesity: meeting both criteria for central obesity and sarcopenia.

Table 3 presents adjusted mean intakes of NOVA food groups by obesity and PS status. Relative to healthy controls, the PS group showed higher intake of PCI (*p* = 0.012) and lower intake of PF (*p* = 0.047). The sarcopenic obesity group showed lower intake of MPF (*p* = 0.046) and higher intake of UPF (*p* = 0.010).

**Table 3:** Intake of NOVA food groups (MPF, PCI, PF, UPF) by four phenotypes defined by abdominal obesity and probable sarcopenia.

	Healthy control^a^	Central obesity^b^	Probable sarcopenia^c^	Sarcopenic obesity^d^	*p*-value
MPF (NOVA 1)	53.8 (1.1)	52.5 (1.3)	55.6 (2.0)	48.7 (2.3)*	0.116
PI (NOVA 2)	0.54 (0.1)	0.39 (0.1)	1.09 (0.2)*	0.70 (0.2)	0.021
PF (NOVA 3)	27.29 (0.7)	26.66 (0.9)	24.18 (1.4)*	27.46 (1.6)	0.236
UPF (NOVA 4)	18.4 (0.8)	20.5 (0.9)	19.1 (1.5)	23.2 (1.7)*	0.051

MPF, minimally processed foods (NOVA 1); PCI, processed culinary ingredients (NOVA 2); PF, processed foods (NOVA 3); UPF, ultra-processed foods (NOVA 4); PA, physical activity. Probable sarcopenia is interchangeably referred to as sarcopenia in this study.

Data are presented as mean (standard deviation). Means were estimated using multiple regression analysis with the healthy control group as the reference, adjusting for age, sex, education level, household income, alcohol consumption, smoking status, and meeting recommended PA levels. *p*-values represent overall group differences from the same model. Total energy intake was adjusted for all covariates except those expressed as %kcal/day.

*p < 0.05 compared with the healthy control group.

a Healthy control: participants without central obesity and sarcopenia.

b Central obesity: waist circumference ≥90 cm for males and ≥85 cm for females.

c Probable sarcopenia: handgrip strength ≤28 kg for males and ≤18 kg for females.

d Sarcopenic obesity: meeting both criteria for central obesity and sarcopenia.

Table 4 shows associations of food intake with odds across groups defined by obesity and PS status. Per-quartile higher total protein intake was associated with lower odds of PS (OR = 0.73, 95% CI: 0.56–0.95), and per-quartile higher PF intake was similarly associated with lower odds of PS (OR = 0.77, 95% CI: 0.59–0.99). In contrast, per-quartile higher UPF intake was associated with higher odds of abdominal obesity (OR = 1.26, 95% CI: 1.03–1.54) and sarcopenic obesity (OR = 1.37, 95% CI: 1.02–1.84). Higher MPF intake (per quartile) showed a borderline association with lower odds of sarcopenic obesity (OR = 0.75, 95% CI: 0.56–1.01).

**Table 4:** Intakes of nutrients and food groups and their odds ratios for central obesity and probable sarcopenia status using multinomial logistic regression models.

	Total protein	Vitamin A	Carotenoids	MPF (NOVA 1)	PCI (NOVA 2)	PF (NOVA 3)	UPF (NOVA 4)
Healthy control^a^	1.00 (REF)	1.00 (REF)	1.00 (REF)	1.00 (REF)	1.00 (REF)	1.00 (REF)	1.00 (REF)
Central obesity^b^	0.95 (0.78–1.16)	1.05 (0.86–1.27)	1.05 (0.87–1.28)	0.92 (0.77–1.12)	0.92 (0.79–1.1.08)	0.98 (0.81–1.19)	1.26 (1.03–1.54)
Probable sarcopenia^c^	0.73 (0.56–0.95)	0.85 (0.66–1.11)	0.85 (0.65–1.10)	1.11 (0.86–1.44)	1.16 (0.94–1.43)	0.77 (0.59–0.99)	1.09 (0.83–1.42)
Sarcopenic obesity^d^	1.07 (0.79–1.44)	0.86 (0.64–1.15)	0.84 (0.63–1.13)	0.75 (0.56–1.01)	1.05 (0.84–1.34)	1.03 (0.77–1.38)	1.37 (1.02–1.84)

MPF, minimally processed foods (NOVA 1); PCI, processed culinary ingredients (NOVA 2); PF, processed foods (NOVA 3); UPF, ultra-processed foods (NOVA 4). Probable sarcopenia is interchangeably referred to as sarcopenia in this study.

Values are expressed as odds ratios (95% confidence intervals). Intakes of each nutrient or food group (%kcal/day) were divided into quartiles and included in the model as continuous variables. Odds ratios were estimated using a multinomial logistic regression model, adjusting for age, sex, education level, household income, alcohol consumption, smoking status, and meeting recommended PA levels.

a Healthy control: participants without central obesity and sarcopenia.

b Central obesity: waist circumference ≥90 cm for males and ≥85 cm for females.

c Probable sarcopenia: handgrip strength ≤28 kg for males and ≤18 kg for females.

d Sarcopenic obesity: meeting both criteria for central obesity and sarcopenia.

## 4 Discussion

This cross-sectional study examined associations between dietary intake and four groups defined by obesity and PS status among adults aged 40–60 years in Gangwon Province. Compared with the healthy control group, the PS group showed lower adjusted mean intakes of protein, vitamin A, and carotenoids, whereas the sarcopenic obesity group showed lower intake of MPF and higher intake of UPF.

In this study, the prevalence of PS and sarcopenic obesity was 13.1% and 9.3%, respectively. A Korean study applying the same criteria reported lower prevalence (PS 8.6%, sarcopenic obesity 6.2%) among adults aged ≥40 years (45), and a UK cohort with an older mean age (76 years) reported PS prevalence ranges of 7.7%–21.1% in men and 5.9%–21.3% in women (46). Despite the younger mean age in our sample (56.0 years), the observed prevalences were comparatively high. This pattern may reflect the rural context of the study population. Previous studies have reported higher prevalence of obesity and PS, as well as less favorable muscle health indicators, in rural versus urban areas (47–49), including higher PS prevalence among rural residents in Korea and India (45, 50).

The proportion of energy from protein was significantly lower in the PS group (13.8%) than in the healthy control (14.7%), obesity (14.6%), and sarcopenic obesity (14.9%) groups. Per-quartile higher total protein intake was associated with lower odds of PS. The average protein energy ratio among participants (14.3%) was below the 2019 national average for Korean adults (15.6%) (42), while remaining within the recommended range of 7%–20% (51). Notably, protein intake in the PS group was lower despite already modest intake at the population level. These findings are consistent with prior reports of associations between higher protein intake and more favorable muscle mass and strength in middle-aged and older adults (52), as well as Korean data reporting associations between lower protein intake, reduced grip strength, and higher PS risk (53).

In this study, the PS group had significantly lower intakes of vitamin A and carotenoids compared with the healthy control group, which is consistent with prior reports on their potential roles in muscle health. Vitamin A’s antioxidant and anti-inflammatory properties may influence chronic inflammation in muscle and adipose tissue, which could be relevant to muscle maintenance (54). Several studies have reported associations between lower circulating carotenoid levels and faster decline in walking speed (55), greater risk of frailty (56), and accelerated loss of muscle strength (57), particularly among females. In the present study, carotenoid intake remained lower in the PS group after adjustment for sex and other covariates, a pattern compatible with a potential role of carotenoids in muscle health across sexes. Given the structural diversity of carotenoids and vitamin A, further work using prospective designs and biomarker-based assessments is needed to clarify compound-specific mechanisms and interactions with protein, particularly in populations with relatively low protein intake, and to strengthen causal inference.

This study found no significant associations between sarcopenia and other macronutrients or micronutrients aside from protein, carotenoids, and vitamin A. Although previous research suggested roles for vitamin D, vitamin C, and omega-3 fatty acids in sarcopenia prevention, findings remain inconsistent or inconclusive (20), potentially due to regional and lifestyle differences. Traditional Korean diets common in rural areas, characterized by high carbohydrate intake from refined grains (58, 59), may be associated with relatively lower protein intake. Such dietary patterns, shared across groups, may partly explain the lack of nutrient intake differences observed between sarcopenia and control groups. Future studies should incorporate biochemical markers to objectively evaluate nutrient status and clarify the complex relationships between diet and muscle health.

In this study, there were significant differences in PCI and PF intake between the healthy control and PS groups. Notably, higher PF intake was associated with lower odds of PS, whereas PCI differed in the opposite direction. PCI primarily includes culinary ingredients such as oils, salt, and sugar, which are rarely consumed independently but shape overall diets through meal preparation and seasoning (36). This interpretation is consistent with prior evidence showing that higher intakes of added sugars and sodium, core PCI components, are associated with lower overall diet quality in adult populations (60, 61). The Brazilian Dietary Guidelines, informed by the NOVA classification system, specifically recommend using PCI in small amounts and in moderation during culinary preparations, as excessive use can render diets nutritionally unbalanced (62). In the Korean context, PF includes traditionally processed fermented foods such as kimchi, which may have neutral or beneficial nutritional effects depending on other dietary components. Specifically, traditional Korean diets rich in fermented foods have been shown to exert beneficial effects on metabolic health, including improvements in blood pressure and glycemic control (63). However, these observations likely reflect regional dietary characteristics and should not be generalized to processed foods as a whole. Although we adjusted for multiple covariates, the cross-sectional design warrants cautious interpretation, and longitudinal studies are needed. Moreover, evidence directly linking PCI or PF intake to muscle-related outcomes remains limited, and further research examining the associations between different NOVA processing categories and muscle health is needed.

Participants with sarcopenic obesity reported higher UPF consumption, consistent with previous findings showing greater UPF intake among individuals with sarcopenic or obese phenotypes (27). However, longitudinal studies examining UPF intake and muscle outcomes have shown inconsistent results. A prospective Chinese cohort found that higher UPF consumption was associated with faster decline in handgrip strength over 3 years (26). Recent evidence from the Framingham Offspring Study also demonstrated that higher UPF intake was associated with increased odds of frailty development and annual declines in grip strength and gait speed over a 10-year follow-up period (64). Despite these findings, a systematic review and meta-analysis reported inconsistent evidence, with cohort studies showing significant associations with frailty but not with muscle strength, while cross-sectional studies indicated associations with low muscle strength (65). Given the cross-sectional design of the present study, reverse causation cannot be ruled out; individuals with sarcopenic obesity may have a greater tendency to choose convenient or ultra-processed foods due to functional limitations or reduced cooking capacity.

In South Korea, the contribution of UPFs to total food intake increased from 23.1% in 2010–2012 to 26.1% in 2016–2018, showing a consistent upward trend across all age and socioeconomic groups (66). In the present study, UPFs accounted for 23.2% of total food intake, comparable to the national level in 2010–2012. Although no significant differences in individual nutrient intakes were observed, the contrasting patterns of MPF and UPF consumption may reflect differences in overall dietary quality related to muscle health. Given the cross-sectional design and the growing public health relevance of UPF consumption, additional prospective studies are warranted to clarify the relationship between UPF intake and muscle-related outcomes.

This study has several limitations that should be considered when interpreting the findings. First, the cross-sectional design does not allow causal inference, and reverse causation cannot be completely ruled out. Second, although this study adjusted for multiple sociodemographic and lifestyle covariates, the possibility of residual confounding cannot be excluded. Third, dietary intake assessed via a self-reported food frequency questionnaire may be subject to recall bias, and the NOVA classification adapted to Korean mixed dishes may not be fully validated. Future studies incorporating objective biomarkers of dietary intake would help validate these findings and reduce measurement error. Fourth, although the overall sample size was adequate for primary analyses, statistical power may have been limited for detecting certain associations in subgroup analyses. Fifth, selection bias may have occurred as participants were community-dwelling volunteers who may differ from non-participants in health status and dietary behaviors, and survival bias cannot be ruled out given that individuals with severe sarcopenia or poor health may have been less likely to participate. Finally, as participants were recruited from rural and mountainous areas in Gangwon Province, the findings may not be generalizable to urban populations or those with different cultural and dietary contexts.

Despite these limitations, this study has several notable strengths. To our knowledge, few studies have examined the relationship between sarcopenic obesity phenotypes and NOVA food processing levels, particularly UPF consumption, among Korean adults aged under 70 years. Using PS instead of clinically defined sarcopenia is meaningful, as early identification of individuals at risk before the onset of clinically diagnosed sarcopenia is essential for prevention and public health interventions. Because the participants were community-dwelling adults under 70 years who generally maintain normal physical function, evaluating PS provides a more appropriate perspective on early metabolic and muscular risk profiles. Taken together, despite its cross-sectional nature, this study provides valuable evidence for understanding the role of dietary processing level in muscle health and may inform future prevention strategies targeting middle-aged and older adult populations.

In this cross-sectional study, lower intakes of protein, vitamin A, and carotenoids, together with higher PCI intake, were associated with higher odds of PS, whereas higher PF intake was associated with lower odds of PS. Higher UPF consumption was linked to greater odds of obesity and sarcopenic obesity, while higher MPF intake showed an inverse association with sarcopenic obesity. These findings provide preliminary evidence that differences in food processing levels may be related to early metabolic and muscle health profiles. Further longitudinal research is needed to confirm these associations and clarify their implications for prevention strategies.

## Data Availability

The datasets presented in this article are not readily available due to the confidentiality agreements and the absence of explicit participant consent for data sharing. Requests to access the datasets should be directed to SP, sopark@hallym.ac.kr; JS, emd76@catholic.ac.kr.

## Group member of GOMS study group, Korean Society for the Study of Obesity

We thank the following contributors to the Gangwon Obesity and Metabolic Syndrome (GOMS) study: Kim Won Jun (Gangneung Asan Hospital), Jeehyun Kang (Konyang University Hospital), Kyoung Kon Kim (Gachon University), Sung Soo Kim (Chungnam National University), Ga Eun Nam (Korea University Guro Hospital), Hyojin Park (Korea University Guro Hospital), Youngeun Seo (Chuncheon National Hospital), Suh Won Yoon (Chungnam National University), Hyun Ju You (Seoul National University), Sang Youl Rhee (Kyung Hee University College of Medicine), Jung Eun Lee (Seoul National University), Joonyub Lee (The Catholic University of Korea), Chang Beom Lee (Hanyang University Guri Hospital), Tae Kyung Lee (Chuncheon National Hospital), Yoon Jeong Cho (Daegu Catholic University School of Medicine), Yang Im Hur (CHA University Bundang Medical Center), and Sangmo Hong (Hanyang University Guri Hospital).

## References

[1] Jeong S-M Jung J-H Yang YS Kim W Cho IY Lee Y-B . 2023 obesity fact sheet: prevalence of obesity and abdominal obesity in adults, adolescents, and children in Korea from 2012 to 2021. J Obes Metab Syndr. (2024) 33:27–35. doi: 10.7570/jomes24012, 38531533 PMC11000515

[2] Lee HA Park H. Comorbidity network analysis related to obesity in middle-aged and older adults: findings from Korean population-based survey data. Epidemiol Health. (2021) 43:e2021018. doi: 10.4178/epih.e2021018, 33677857 PMC8060529

[3] Yang YS Han B-D Han K Jung J-H Son JW. Obesity fact sheet in Korea, 2021: trends in obesity prevalence and obesity-related comorbidity incidence stratified by age from 2009 to 2019. J Obes Metab Syndr. (2022) 31:169. doi: 10.7570/jomes22024, 35770450 PMC9284570

[4] Yuan L Chang M Wang J. Abdominal obesity, body mass index and the risk of frailty in community-dwelling older adults: a systematic review and meta-analysis. Age Ageing. (2021) 50:1118–28. doi: 10.1093/ageing/afab039, 33693472

[5] De StefanoF ZambonS GiacomettiL SergiG CortiM ManzatoE . Obesity, muscular strength, muscle composition and physical performance in an elderly population. J Nutr Health Aging. (2015) 19:785–91. doi: 10.1007/s12603-015-0482-3, 26193864

[6] Di Iorio A Di Blasio A Napolitano G Ripari P Paganelli R Cipollone F. High fat mass, low muscle mass, and arterial stiffness in a population of free-living healthy subjects: the “al passo con la tua salute” project. Medicine. (2019) 98:e16172. doi: 10.1097/MD.0000000000016172, 31261548 PMC6616375

[7] Choi J-Y Kim K-i. Diagnosis and management of sarcopenia. J Korean Med Assoc. (2024) 67:461–6. doi: 10.5124/jkma.2024.67.7.461

[8] Kim SH Jeong JB Kang J Ahn D-W Kim JW Kim BG . Association between sarcopenia level and metabolic syndrome. PLoS One. (2021) 16:e0248856. doi: 10.1371/journal.pone.0248856, 33739984 PMC7978348

[9] Janssen I Heymsfield SB Ross R. Low relative skeletal muscle mass (sarcopenia) in older persons is associated with functional impairment and physical disability. J Am Geriatr Soc. (2002) 50:889–96. doi: 10.1046/j.1532-5415.2002.50216.x, 12028177

[10] Chang SF Lin PL. Systematic literature review and meta-analysis of the association of sarcopenia with mortality. Worldviews Evid Based Nurs. (2016) 13:153–62. doi: 10.1111/wvn.12147, 26844538

[11] Cruz-Jentoft AJ Baeyens JP Bauer JM Boirie Y Cederholm T Landi F . Sarcopenia: European consensus on definition and diagnosis: report of the European working group on sarcopenia in older people. Age Ageing. (2010) 39:412–23. doi: 10.1093/ageing/afq034, 20392703 PMC2886201

[12] Atkins JL Wannamathee SG. Sarcopenic obesity in ageing: cardiovascular outcomes and mortality. Br J Nutr. (2020) 124:1102–13. doi: 10.1017/S0007114520002172, 32616084

[13] MolinoS DossenaM BuonocoreD VerriM. Sarcopenic obesity: an appraisal of the current status of knowledge and management in elderly people. J Nutr Health Aging. (2016) 20:780–8. doi: 10.1007/s12603-015-0631-8, 27499312

[14] Jiang M Ren X Han L Zheng X. Associations between sarcopenic obesity and risk of cardiovascular disease: a population-based cohort study among middle-aged and older adults using the CHARLS. Clin Nutr. (2024) 43:796–802. doi: 10.1016/j.clnu.2024.02.002, 38350287

[15] Zhang X Xie X Dou Q Liu C Zhang W Yang Y . Association of sarcopenic obesity with the risk of all-cause mortality among adults over a broad range of different settings: a updated meta-analysis. BMC Geriatr. (2019) 19:1–14. doi: 10.1186/s12877-019-1195-y, 31269909 PMC6610788

[16] Wannamethee SG Atkins JL. Sarcopenic obesity and cardiometabolic health and mortality in older adults: a growing health concern in an ageing population. Curr Diab Rep. (2023) 23:307–14. doi: 10.1007/s11892-023-01522-2, 37566368 PMC10640508

[17] Marzetti E Calvani R Tosato M Cesari M Di Bari M Cherubini A . Sarcopenia: an overview. Aging Clin Exp Res. (2017) 29:11–7. doi: 10.1007/s40520-016-0704-5, 28155183

[18] Rizzoli R Reginster J-Y Arnal J-F Bautmans I Beaudart C Bischoff-Ferrari H . Quality of life in sarcopenia and frailty. Calcif Tissue Int. (2013) 93:101–20. doi: 10.1007/s00223-013-9758-y, 23828275 PMC3747610

[19] Hill JO Peters JC. Environmental contributions to the obesity epidemic. Science. (1998) 280:1371–4. doi: 10.1126/science.280.5368.1371, 9603719

[20] Ganapathy A Nieves JW. Nutrition and sarcopenia—what do we know? Nutrients. (2020) 12:1755. doi: 10.3390/nu12061755, 32545408 PMC7353446

[21] Monteiro CA Cannon G Moubarac JC Levy RB Louzada MLC Jaime PC. The UN decade of nutrition, the NOVA food classification and the trouble with ultra-processing. Public Health Nutr. (2018) 21:5–17. doi: 10.1017/S1368980017000234, 28322183 PMC10261019

[22] Tran NR Leech RM Livingstone KM McNaughton SA. Achieving high diet quality at eating occasions: findings from a nationally representative study of Australian adults. Br J Nutr. (2024) 131:868–79. doi: 10.1017/S0007114523002325, 37855251 PMC10864991

[23] Carbonneau E Lamarche B Provencher V Desroches S Robitaille J Vohl M-C . Liking for foods high in salt and fat is associated with a lower diet quality but liking for foods high in sugar is not–results from the PREDISE study. Food Qual Prefer. (2021) 88:104073. doi: 10.1016/j.foodqual.2020.104073

[24] Dekker LH Vinke PC Riphagen IJ Minović I Eggersdorfer ML van den Heuvel EG . Cheese and healthy diet: associations with incident cardio-metabolic diseases and all-cause mortality in the general population. Front Nutr. (2019) 6:185. doi: 10.3389/fnut.2019.00185, 31921878 PMC6927928

[25] HaoJ ZhouP QiuH. Association between ultra-processed food consumption and frailty in American elder people: evidence from a cross-sectional study. J Nutr Health Aging. (2022) 26:688–97. doi: 10.1007/s12603-022-1824-6, 35842759

[26] Zhang S Gu Y Rayamajhi S Thapa A Meng G Zhang Q . Ultra-processed food intake is associated with grip strength decline in middle-aged and older adults: a prospective analysis of the TCLSIH study. Eur J Nutr. (2022) 61:1331–41. doi: 10.1007/s00394-021-02737-3, 34791509

[27] Kong W Xie Y Hu J Ding W Cao C. Higher ultra processed foods intake is associated with low muscle mass in young to middle-aged adults: a cross-sectional NHANES study. Front Nutr. (2024) 11:1280665. doi: 10.3389/fnut.2024.1280665, 38439924 PMC10909937

[28] Sarcopenia IWGo. Sarcopenia: an undiagnosed condition in older adults. Current consensus definition: prevalence, etiology, and consequences. J Am Med Dir Assoc. (2011) 12:249. doi: 10.1016/j.jamda.2011.01.003 21527165 PMC3377163

[29] Marcell TJ. Sarcopenia: causes, consequences, and preventions. J Gerontol A Biol Sci Med Sci. (2003) 58:M911–6. doi: 10.1093/gerona/58.10.m911 14570858

[30] Cruz-Jentoft AJ Bahat G Bauer J Boirie Y Bruyère O Cederholm T . Sarcopenia: revised European consensus on definition and diagnosis. Age Ageing. (2019) 48:16–31. doi: 10.1093/ageing/afy169, 30312372 PMC6322506

[31] Korea S. 2021 Statistics on the elderly. Daejeon: Statistics Korea (2021).

[32] News K. Gangwon Province ‘obesity rate’ ranks second nationwide. High in Cheorwon and Hwacheon (in Korean): KBS News (2024).

[33] Cho YJ Park S Kim SS Park HJ Son JW Lee TK . The Gangwon obesity and metabolic syndrome study: methods and initial baseline data. J Obes Metab Syndr. (2022) 31:303–12. doi: 10.7570/jomes22064, 36581590 PMC9828700

[34] Yun SH Shim J-S Kweon S Oh K. Development of a food frequency questionnaire for the Korea National Health and nutrition examination survey: data from the fourth Korea National Health and nutrition examination survey (KNHANES IV). Korean J Nutr. (2013) 46:186–96. doi: 10.4163/kjn.2013.46.2.186

[35] Kim DW Song S Lee JE Oh K Shim J Kweon S . Reproducibility and validity of an FFQ developed for the Korea National Health and nutrition examination survey (KNHANES). Public Health Nutr. (2015) 18:1369–77. doi: 10.1017/S1368980014001712, 25167205 PMC10271806

[36] Monteiro CA Cannon G Levy RB Moubarac J-C Louzada ML Rauber F . Ultra-processed foods: what they are and how to identify them. Public Health Nutr. (2019) 22:936–41. doi: 10.1017/S1368980018003762, 30744710 PMC10260459

[37] Park HJ Park S Kim JY. Development of Korean NOVA food classification and estimation of ultra-processed food intake among adults: using 2018 Korea National Health and nutrition examination survey. Korean J Community Nutr. (2022) 27:455–67. doi: 10.5720/kjcn.2022.27.6.455

[38] Jung S Park S Kim JY. Comparison of dietary share of ultra-processed foods assessed with a FFQ against a 24-h dietary recall in adults: results from KNHANES 2016. Public Health Nutr. (2022) 25:1166–75. doi: 10.1017/S1368980022000179, 35042567 PMC9991629

[39] Han K. Characteristics of obesity in elderly and approaching for diagnosis. J Geriatr Neurol. (2022) 1:59–64. doi: 10.53991/jgn.2022.00101

[40] Després J-P Lemieux I Bergeron J Pibarot P Mathieu P Larose E . Abdominal obesity and the metabolic syndrome: contribution to global cardiometabolic risk. Arterioscler Thromb Vasc Biol. (2008) 28:1039–49. doi: 10.1161/ATVBAHA.107.159228, 18356555

[41] Janssen I Katzmarzyk PT Ross R. Waist circumference and not body mass index explains obesity-related health risk. Am J Clin Nutr. (2004) 79:379–84. doi: 10.1093/ajcn/79.3.379, 14985210

[42] Lee SY Park HS Kim DJ Han JH Kim SM Cho GJ . Appropriate waist circumference cutoff points for central obesity in Korean adults. Diabetes Res Clin Pract. (2007) 75:72–80. doi: 10.1016/j.diabres.2006.04.013, 16735075

[43] Chen L-K Liu L-K Woo J Assantachai P Auyeung T-W Bahyah KS . Sarcopenia in Asia: consensus report of the Asian working Group for Sarcopenia. J Am Med Dir Assoc. (2014) 15:95–101. doi: 10.1016/j.jamda.2013.11.025, 24461239

[44] Kim Y Yang Y Park H Kim J. Development of physical activity guidelines and self-prescription guides for Korean. Seoul: Korea Health Promotion Institute (2012).

[45] Lee H Park S. Regional differences in the associations of diet quality, obesity, and possible sarcopenia using the seventh Korea National Health and nutrition examination survey (2016-2018). Epidemiol Health. (2023) 45:45. doi: 10.4178/epih.e2023059, 37402414 PMC10667583

[46] Kilgour A Redmond P Taylor A Deary IJ Starr J Shenkin SD. Prevalence of sarcopenia in a longitudinal UK cohort study using EWGSOP2 criteria varies widely depending onwhich measures of muscle strength and performance are used. Age Ageing. (2020) 49:i22–3. doi: 10.1093/ageing/afz188.01

[47] Okobi OE Ajayi OO Okobi TJ Anaya IC Fasehun OO Diala CS . The burden of obesity in the rural adult population of America. Cureus. (2021) 13:e15770. doi: 10.7759/cureus.15770, 34295580 PMC8290986

[48] Keramat SA Alam K Al-Hanawi MK Gow J Biddle SJ Hashmi R. Trends in the prevalence of adult overweight and obesity in Australia, and its association with geographic remoteness. Sci Rep. (2021) 11:11320. doi: 10.1038/s41598-021-90750-1, 34059752 PMC8166878

[49] Aziz J Reid K Batsis J Fielding R. Urban-rural differences in the prevalence of muscle weakness and slow gait speed: a cross-sectional analysis from the NHANES (2001-2002 and 2011-2014). JAR Life. (2021) 10:19–25. doi: 10.14283/jarlife.2021.4, 35783581 PMC9248630

[50] Bhat G Ireland A Shah N Gondhalekar K Mandlik R Kajale N . Prevalence and factors associated with sarcopenia among urban and rural Indian adults in middle age: a cross-sectional study from Western India. PLOS Glob Public Health. (2024) 4:e0003553. doi: 10.1371/journal.pgph.0003553, 39352920 PMC11444403

[51] Kim E Chung S Hwang J-T Park YJ. 2020 Korean dietary reference intakes for protein: estimation of protein requirements and the status of dietary protein intake in the Korean population. J Nutr Health. (2022) 55:10–20. doi: 10.4163/jnh.2022.55.1.10

[52] Coelho-Junior HJ Calvani R Azzolino D Picca A Tosato M Landi F . Protein intake and sarcopenia in older adults: a systematic review and meta-analysis. Int J Environ Res Public Health. (2022) 19:8718. doi: 10.3390/ijerph19148718, 35886571 PMC9320473

[53] Han M Woo K Kim K. Association of Protein Intake with sarcopenia and related indicators among Korean older adults: a systematic review and Meta-analysis. Nutrients. (2024) 16:4350. doi: 10.3390/nu16244350, 39770971 PMC11677379

[54] Palace VP Khaper N Qin Q Singal PK. Antioxidant potentials of vitamin a and carotenoids and their relevance to heart disease. Free Radic Biol Med. (1999) 26:746–61. doi: 10.1016/S0891-5849(98)00266-4, 10218665

[55] Alipanah N Varadhan R Sun K Ferrucci L Fried L Semba RD. Low serum carotenoids are associated with a decline in walking speed in older women. J Nutr Health Aging. (2009) 13:170–5. doi: 10.1007/s12603-009-0053-6, 19262947 PMC2748676

[56] Semba RD Bartali B Zhou J Blaum C Ko C-W Fried LP. Low serum micronutrient concentrations predict frailty among older women living in the community. J Gerontol Ser A Biol Med Sci. (2006) 61:594–9. doi: 10.1093/gerona/61.6.594, 16799142

[57] Lauretani F Semba RD Bandinelli S Dayhoff-Brannigan M Giacomini V Corsi AM . Low plasma carotenoids and skeletal muscle strength decline over 6 years. J Gerontol Ser A Biol Med Sci. (2008) 63:376–83. doi: 10.1093/gerona/63.4.376, 18426961 PMC4101895

[58] Song S Lee JE Song WO Paik H-Y Song Y. Carbohydrate intake and refined-grain consumption are associated with metabolic syndrome in the Korean adult population. J Acad Nutr Diet. (2014) 114:54–62. doi: 10.1016/j.jand.2013.08.025, 24200655

[59] Kim S Moon S Popkin BM. The nutrition transition in South Korea. Am J Clin Nutr. (2000) 71:44–53. doi: 10.1093/ajcn/71.1.44, 10617945

[60] Cara KC Fan Z Chiu Y-H Jiang X Alhmly HF Chung M. Associations between intake of dietary sugars and diet quality: a systematic review of recent literature. Nutrients. (2024) 16:1549. doi: 10.3390/nu16111549, 38892483 PMC11174080

[61] Mercado CI Cogswell ME Perrine CG Gillespie C. Diet quality associated with total sodium intake among US adults aged ≥18 years—national health and nutrition examination survey, 2009–2012. Nutrients. (2017) 9:1164. doi: 10.3390/nu9111164, 29068397 PMC5707636

[62] Monteiro CA Cannon G Moubarac J-C Martins APB Martins CA Garzillo J . Dietary guidelines to nourish humanity and the planet in the twenty-first century. A blueprint from Brazil. Public Health Nutr. (2015) 18:2311–22. doi: 10.1017/S1368980015002165, 26205679 PMC10271430

[63] Jung S-J Park S-H Choi E-K Cha Y-S Cho B-H Kim Y-G . Beneficial effects of Korean traditional diets in hypertensive and type 2 diabetic patients. J Med Food. (2014) 17:161–71. doi: 10.1089/jmf.2013.3042, 24456367 PMC3901348

[64] Konieczynski EM Sahni S Jacques PF Naumova EN. Ultra-processed food and frailty: evidence from a prospective cohort study and implications for future research. Nutrients. (2025) 17:2631. doi: 10.3390/nu17162631, 40871659 PMC12389690

[65] Hojjati Kermani MA Awlqadr FH Talebi S Mehrabani S Camera DM Bagheri R . The association of ultra-processed food intake on age-related muscle conditions: a systematic review and dose–response meta-analysis with meta-regression. J Health Popul Nutr. (2025) 44:271. doi: 10.1186/s41043-025-00986-0, 40739278 PMC12312346

[66] Shim J-S Shim S-Y Cha H-J Kim J Kim HC. Socioeconomic characteristics and trends in the consumption of ultra-processed foods in Korea from 2010 to 2018. Nutrients. (2021) 13:1120. doi: 10.3390/nu13041120, 33805412 PMC8065678

