# Supplementary material for: Comparison of nutrients and ultra-processed food consumption between different phenotypes defined by abdominal obesity and sarcopenia
Source: Front Nutr. 2026 Jan 19;12:1683196. doi: 10.3389/fnut.2025.1683196 (PMC12863201; doi:10.3389/fnut.2025.1683196)
Supplement: Supplementary file 1 [file Table_1.docx]

**Supplementary Material**

**Comparison of Nutrients and Ultra-Processed Food Consumption Between Different Phenotypes Defined by Abdominal Obesity and Sarcopenia**

**Eunjin Jang1, Sarang Jeong1, Jinhyun Kim1,2, Sukyoung Jung3, Jee Young Kim4, Jung Eun Lee5, Sohyun Park1,2*, Jang Won Son6***

**On behalf of the GOMS study group, Korean Society for the Study of Obesity**

**Sohyun Park1,2*, Jang Won Son6***

***These authors share corresponding authorship.**

**Supplementary Table 1. Anthropometric and metabolic characteristics of participants by abdominal obesity and probable sarcopenia**

**Supplementary Table 1. Anthropometric and metabolic characteristics of participants by abdominal obesity and probable sarcopenia**

|  | **Healthy control^a^** | **Central obesity^b^** | **Probable sarcopenia^c^** | **Sarcopenic obesity^d^** | **P - value** |
| --- | --- | --- | --- | --- | --- |
| BMI(kg/m2)^e^ | 22.5 ± 2.3 | 27.9 ± 3.2 | 23.0 ± 2.2 | 27.4 ± 3.3 | < 0.001 |
| Waist circumference (cm)^e^ | 77.8 ± 6.7 | 93.7 ± 6.9 | 77.9 ± 5.8 | 92.6 ± 6.8 | < 0.001 |
| Mean handgrip strength (kg)^e^ | 26.9 ± 7.5 | 28.1 ± 7.7 | 16.4 ± 3.8 | 16.9 ± 4.3 | < 0.001 |
| Metabolic syndrome components^e^ |  |  |  |  |  |
| High triglycerides (n =533) |  |  |  |  | < 0.001 |
| No | 166.0 (69.8) | 81.0 (46.0) | 49.0 (70.0) | 23.0 (46.9) |  |
| Yes | 72.0 (30.3) | 95.0 (54.0) | 21.0 (30.0) | 26.0 (53.1) |  |
| Low HDL cholesterol (n = 534) |  |  |  |  | 0.003 |
| No | 199.0 (83.6) | 120.0 (68.2) | 52.0 (74.3) | 36.0 (72.0) |  |
| Yes | 39.0 (16.4) | 56.0 (31.8) | 18.0 (25.7) | 14.0 (28.0) |  |
| High blood pressure |  |  |  |  | < 0.001 |
| No | 105.0 (43.9) | 37.0 (21.0) | 30.0 (42.9) | 15.0 (30.0) |  |
| Yes | 134.0 (56.1) | 139.0 (79.0) | 40.0 (57.1) | 35.0 (70.0) |  |
| High fasting glucose (n=533) |  |  |  |  | < 0.001 |
| No | 150.0 (63.0) | 68.0 (38.6) | 52.0 (74.3) | 28.0 (57.1) |  |
| Yes | 88.0 (37.0) | 108.0 (61.4) | 18.0 (25.7) | 21.0 (42.9) |  |
| Number of metabolic syndrome components present (n =533) |  |  |  |  | < 0.001 |
| 0 | 53.0 (22.3) | 13.0 (7.4) | 18.0 (25.7) | 6.0 (12.2) |  |
| 1 | 83.0 (34.9) | 27.0 (15.3) | 24.0 (34.3) | 13.0 (26.5) |  |
| 2 | 63.0 (26.5) | 57.0 (32.4) | 14.0 (20.0) | 13.0 (26.5) |  |
| 3 | 33.0 (13.9) | 59.0 (33.5) | 11.0 (15.7) | 11.0 (22.5) |  |
| 4 | 6.0 (2.5) | 20.0 (11.4) | 3.0 (4.3) | 6.0 (12.2) |  |

Abbreviations: HDL, high-density lipoprotein; PA, physical activity. Abdominal obesity and probable sarcopenia are referred to as central obesity and sarcopenia in this study.

^a^ Healthy control: participants without central obesity and sarcopenia.

^b^ Central obesity group: waist circumference ≥90 cm for males and ≥85 cm for females.

^c^ Sarcopenia group: handgrip strength ≤28 kg for males and ≤18 kg for females.

^d^ Sarcopenic obesity group: meeting both criteria for central obesity and sarcopenia.

^e^ Adjusted for age and sex.

Definitions of metabolic syndrome components:

High triglycerides were defined as triglyceride levels ≥150 mg/dL.

Low HDL cholesterol was defined as <40 mg/dL for males and <50 mg/dL for females.

High blood pressure was defined as systolic blood pressure ≥130 mmHg or diastolic blood pressure ≥85 mmHg.

High fasting glucose was defined as fasting plasma glucose ≥100 mg/dL.

Continuous variables are presented as mean ± standard error; categorical variables are presented as n (%).

^*^ P-values were calculated using linear regression for continuous variables and chi-square test for categorical variables.

**Supplementary Table 2. Categorization of food and beverage items from the semiquantitative food frequency questionnaire into NOVA groups**

| Food item | NOVA group assignment |
| --- | --- |
| White rice | MPF |
| Barley, mixed grains | MPF |
| Bibimbap, fried rice | PF |
| Gimbap | PF |
| Curry with rice | UPF |
| Instant noodles | UPF |
| Hot noodles | UPF |
| Black bean sauce noodles | UPF |
| Cold noodles | UPF |
| Rice cake soup | PF |
| Dumplings | UPF |
| White breads | UPF |
| Butter, margarine | PCI |
| Jam | UPF |
| Sweet red bean bread, other breads with sweet red bean, other breads with cream | UPF |
| Castella, cake, chocolate pie | UPF |
| Pizza | UPF |
| Hamburger, sandwich | UPF |
| Rice cake, other rice cakes | UPF |
| Stir-fried rice cakes (Tteokbokki) | UPF |
| Cereals | UPF |
| Ox bone soup (Sullungtang), Gomguk/Gomtang, beef bone soup | PF |
| Pork backbone stew (Gamjatang) | PF |
| Boiled mud-fish soup (Chueotang) | PF |
| Spicy fish stew (Maeuntang) | PF |
| Seaweed soup | PF |
| Beef and radish soup, spicy beef soup (Yukgaejang), radish soup | PF |
| Dried pollock soup (Bugeoguk) | PF |
| Bean paste soup | PF |
| Bean paste stew, extra strong fermented soybean paste stew | PF |
| Kimchi stew, stir-fried kimchi | PF |
| Army soup | UPF |
| Tofu stew, spicy soft tofu stew | PF |
| Tofu, spicy braised tofu, soy sauce braised tofu, pan fried tofu | PF |
| Beans/beans cooked in soy sauce | PF |
| Fried eggs, egg roll | PF |
| Boiled eggs, steamed eggs | PF |
| Grilled pork belly | MPF |
| Boiled or steamed pork with soybean paste | PF |
| Spicy stir-fried pork, spicy pork Bulgogi, grilled pork rib, steamed pork rib | PF |
| Fried pork, pork cutlet | PF |
| Grilled beef | PF |
| Grilled marinated beef (Korean style beef Bulgogi) | PF |
| Processed meat | UPF |
| Korean blood sausage | UPF |
| Ginseng chicken soup (Samgye-tang) | PF |
| Stir-fried spicy chicken (Dak Galbi), spicy Korean chicken stew | PF |
| Fried chicken | PF |
| Grilled marinated duck | PF |
| Grilled or braised mackerel, grilled or braised pacific saury | PF |
| Grilled or braised belt fish, grilled or braised corbina | PF |
| Anchovy, stir-fried anchovy | PF |
| Squid, stir-fried or seasoned dried shredded squid, dried squid | PF |
| Marinated raw crab | PF |
| Salted shrimp, salted squid, salted clamp | PF |
| Fishcake | UPF |
| Bean sprouts (marinated, soup), mung bean sprouts (marinated) | PF |
| Spinach (marinated) | PF |
| Bellflower root (marinated) | PF |
| Zucchini (marinated, pancake) | PF |
| Stir-fried fern brake/bracken, seasoned aster, seasoned eggplant, and other seasoned vegetables | PF |
| Cucumber (marinated) | PF |
| Radish (marinated, picked, dried) | PF |
| Vegetable salad with dressing | PF |
| Seasoned green onion, seasoned chive | PF |
| Ssam (lettuce, perilla leaves, nappa cabbage, zucchini leaves), green peppers | MPF |
| Steamed broccoli, steamed cabbage | MPF |
| Garlic | MPF |
| Soybean paste mixed with red pepper paste | UPF |
| Cabbage kimchi | PF |
| Other kimchi | PF |
| Pickled vegetable (green pepper, garlic, perilla leaves, onion, radish), pickled cucumber | PF |
| Braised lotus roots, braised boiled burdock | PF |
| Korean pancake (chive, kimchi) | PF |
| Stir-fried noodles and vegetables | UPF |
| Stir-fried mushrooms | PF |
| Grilled laver, laver, marinated laver | PF |
| Seasoned sea lettuce, seaweed with vinegar seasoning | PF |
| Stir-fried seaweed stem | PF |
| Stir-fried potatoes, braised potatoes | PF |
| Steamed potatoes, grilled potatoes | MPF |
| Sweet potato (steamed or grilled) | MPF |
| Corn (steamed or grilled with salt or sugar) | PF |
| Milk | MPF |
| Yogurt (liquid) | UPF |
| Yogurt (solid) | UPF |
| Soybean milk | UPF |
| Strawberry | MPF |
| Tomato, cherry tomato | MPF |
| Oriental melon | MPF |
| Watermelon | MPF |
| Peach | MPF |
| Grape | MPF |
| Apple | MPF |
| Pear | MPF |
| Persimmon/dried persimmon | MPF |
| Tangerine | MPF |
| Banana | MPF |
| Orange | MPF |
| Kiwi | MPF |
| Coffee | MPF |
| Prim | UPF |
| Sugar | UPF |
| Green tea | MPF |
| Soft drink (coke/cider/fruit carbonate beverage) | UPF |
| Fruit juice | UPF |
| Mixed grain powder drink (misu-garu), traditional sweet Korean rice beverage | UPF |
| Snacks | UPF |
| Cookie/cracker | UPF |
| Chocolate | UPF |
| Ice cream | UPF |
| Peanuts | PF |
| Chestnuts | MPF |

Abbreviations: MPF, minimally processed foods; PCI, processed culinary ingredients; PF, processed foods; UPF, ultra-processed foods.
